# Thermal Aging Degradation of High-Viscosity Asphalt Based on Rheological Methods

**DOI:** 10.3390/ma16186250

**Published:** 2023-09-17

**Authors:** Siyue Zhu, Xiantao Qin, Menghui Liao, Yuxi Ma, Hao Xu, Jingyi Chen, Haobo Gao

**Affiliations:** 1 School of Civil Engineering and Architecture, Wuhan Polytechnic University, 68 Xuefu South Road, Wuhan 430023, China; 2 School of Transportation and Logistics Engineering, Wuhan University of Technology, 1178 Heping Avenue, Wuhan 430063, China

**Keywords:** porous asphalt pavement, high-viscosity asphalt, rheological property, thermal aging

## Abstract

With the acceleration of the construction of sponge cities in China, porous asphalt pavement (PA) is has been widely used. High-viscosity asphalt (HVA) is the core material in building PA because it has good rheology properties, which can provide good raveling and rutting resistance. However, due to the open-graded structure of PA, HVA was more susceptible to rapid aging, which significantly affects the durability of PA. To investigate the thermal aging effect on the rheological properties of self-modified HVA (SHVA), five types of asphalts were aged using a rolling thin film oven (RTFO) and pressure aging vessel (PAV). Then, rheological tests were adopted, such as temperature sweep test (TS), repeated creep and recovery test (RCR), and bending beam rheometer test (BBR). The results indicate that during the aging process, the oxidation-induced hardening effect of neat asphalt and the degradation-induced softening effect of the modifier changes the rheology properties of HVA significantly. As the aging progresses, the contribution of the modifiers of HVA to anti-aging performance is greatly reduced. At high temperatures, HVA demonstrates better anti-aging performance than conventional styrene–butadiene–styrene (SBS)-modified asphalt (Guo Chuang, GC). The change of the high-temperature rheological indices of the two HVA types (SHVA and TAFPACK-super HVA (TPS)) showed a smaller activation energy index (EAI), a more considerable viscous component of binder creep stiffness (Gv), and more minor accumulated stain (*r_acc_*), indicating a more significant anti-short-term and long-term aging performance, which is beneficial to the high-temperature performance of asphalts. However, the changes in low-temperature rheological properties do not align with those in high-temperature rheological properties after long-term aging. The BBR test results reveal that TPS exhibits worse low-temperature performance than GC and SHVA. During the thermal aging process, the contribution rate of the modifiers in SHVA against RTFO and PAV aging is higher than that of the modifiers in TPS, which contributes to the superior anti-aging property. Overall, SHVA demonstrates the best anti-aging performance among the five asphalts tested.

## 1. Introduction

Waterlogging has emerged as a significant issue in China, impacting transportation and the ecological environment in recent decades [1,2]. To tackle this problem, China initiated the construction of “sponge cities” in 2015. Porous asphalt pavement, with a void content of approximately 20%, plays a crucial role as an eco-friendly road technology in realizing the concept of a “sponge city” [3]. This innovative pavement also effectively drains surface water and offers additional benefits, such as reducing traffic noise, enhancing skid resistance, and improving driving safety and comfort [4,5]. To enhance the overall road properties of PA, modified asphalt is commonly used due to its open-graded structure and large void content. However, in China’s climate and heavy traffic conditions, conventional SBS-modified asphalt may not guarantee the durability of the bond between the aggregates [6]. This can lead to raveling, water damage, and rutting. As a result, HVA is often employed in PA in China [7]. HVA, with a dynamic viscosity of over 2000 Pa·s, helps to improve the bond between the asphalt and aggregates, enhancing the pavement’s overall performance.

However, the high viscosity of HVA necessitates higher mixing and paving temperatures compared to conventional SBS-modified asphalt mixtures. Specifically, the mixing temperature ranges from 180 to 190 °C, while the paving temperature ranges from 170 to 185 °C [8]. Unfortunately, these elevated temperatures can accelerate the aging process of the HVA. Moreover, large voids in PA make the HVA more susceptible to rapid aging. This susceptibility is primarily due to increased exposure to external factors such as moisture, temperature, and ultraviolet radiation [9]. Consequently, the asphalt becomes progressively brittle and stiff, leading to cohesion failure at high temperatures, adhesion failure at low temperatures, and moisture damage. These issues significantly impact the durability and driving comfort and increase the maintenance cost of PA. Field studies have demonstrated that the service life of PA is noticeably shorter, typically around 10–12 years, compared to ordinary pavement, which can last approximately 18 years [10].

Traditional research has primarily focused on optimizing the formula and studying the road performance of HVA, yielding fruitful results [11,12,13]. These studies have greatly improved properties such as storage stability, viscosity, toughness, and tenacity of HVA, enhancing the road performance of PA. However, the durability of PA remains unsatisfactory [14]. The aging performance of HVA plays a crucial role in extending the durability of PA. Researchers are now paying more attention to the aging properties of HVA. Some studies employ microscopic tests to investigate the aging behavior of polymers in HVA. For example, Sun suggested that the aging characteristics of HVA were related to dual effects and polymer molecular distribution [15]. Hu investigated the microevolution of bitumen microstructure, polymer phase, and polymer–bitumen interaction in HVA [16]. In addition to microstructure, rheological methods are utilized to assess the aging performance of HVA, as its aging performance is intricately linked to changes in its rheological properties. Jhony Habbouche examined the effects of HVA aging by fitting the complex shear modulus (G*) and phase angle (δ) master curves across various temperatures [17]. Similarly, Wang conducted a temperature sweep test (TS) to study the rheological attributes of aged HVA, analyzing the variations in δ and G* with aging. The findings suggest that δ can depict the alterations in HVA throughout the aging process. However, this method was inappropriate for comparing the high-temperature performances of different types of aged HVA [18]. Qurashi, Irfan A recommended employing a rotational viscometer across a temperature spectrum to determine the viscous flow activation energy, which can accurately quantify the aging characteristics of asphalt binder [19]. However, this approach was deemed unsuitable for HVA [20]. Therefore, more suitable indices are needed in order to evaluate and compare the aging resistance of HVA. Additionally, aging will lead to the deterioration of asphalt performance at low temperature, and bending beam rheometer (BBR) testing is the most efficient way to capture the aging effect on low-temperature properties of HVA [21].

Moreover, as the severity of aging increases, HVA becomes more susceptible to permanent deformation. The repeated creep recovery (RCR) and multiple stress creep recovery (MSCR) tests are considered more accurate for assessing asphalt’s resistance to permanent deformation, particularly for modified asphalts. Consequently, a growing body of research employs these tests to investigate the impact of aging on HVA. For example, Hu utilized MSCR tests to evaluate the rheological properties of aged HVA [8]. The primary distinction between RCR and MSCR tests lies in their methodology. RCR applies a constant shear stress, such as 0.1 kPa, over 100 cycles, whereas MSCR employs two different shear stresses (0.1 kPa and 3.2 kPa) over 10 cycles. Given that HVA exhibits high flow resistance and robust deformation recovery, it is less likely to reach the fatigue point in a limited number of cycles. Therefore, RCR appears to be a more suitable method for capturing changes in the high-temperature properties of HVA under varying aging conditions.

In our previous study, which focused on improving the road performance of porous asphalt, we meticulously determined the material composition of SHVA. This involved selecting the most suitable type of neat asphalt and precisely defining the type and dosage of each modifier, encompassing polymers, compatibilizers, and stabilizers [22]. In addition, the formula for SHVA has been continuously refined. We also examined the thermal aging degradation of basic properties of SHVA, including penetration, ductility, and zero-shear viscosity (ZSV) toughness [23]. Our results showed that SHVA had a better resistance to thermal aging than TPS, although the difference was subtle. Therefore, it is imperative to conduct further investigations into the thermal aging behavior of SHVA. SHVA is a typical composite material with a high polymer content, and the type and amount of polymer present are greater than those in conventional SBS-modified asphalts. During thermal aging, the interplay between asphalt oxidation and polymer degradation becomes increasingly intricate compared to SBS-modified asphalts [24]. This added complexity will likely result in variations in SHVA’s rheological properties as it ages. Employing rheological methods to isolate the contributions of neat asphalt and modifiers at various aging stages can help to improve the aging resistance of SHVA and further optimize the material composition of SHVA.

This paper aims to investigate the thermal aging degradation of SHVA based on the rheological method. In addition, we study the effect of base asphalt aging and polymer degradation under different aging statements. Five types of asphalts were selected, including two kinds of neat asphalts for preparing SHVA and TAFPACK Super high-viscosity asphalt (one of the best high-viscosity asphalt modifiers, TPS), as well as conventional SBS-modified asphalts (GC). The asphalts underwent RTFO and PAV aging tests to simulate short-term and long-term aging. Based on the above literature review, both the TS and the RCR tests were chosen to investigate the impact of thermal aging on the high-temperature performance of SHVA. The activation energy index (EAI) derived from the TS test was also introduced. For assessing low-temperature rheological properties, the BBR test was employed. Subsequently, a comparative study was undertaken to quantitatively evaluate the rheological properties of thermally aged HVA.

## 2. Materials and Test Methods

### 2.1. Raw Materials

Neat asphalt SK70 (SK Holdings, Seoul, Republic of Korea) was chosen to prepare SHVA. To facilitate meaningful comparisons, we had previously determined the optimal base asphalt type and the appropriate dosage of the TPS high-viscosity modifier. For the preparation of TPS, we used AS70 (ExxonMobil, Singapore) with a 12% TPS high-viscosity modifier (Taiyu Co., Ltd., Osaka, Japan) by mass of neat asphalt; we also incorporated a commercial product, GC SBS-modified asphalt (Hubei Guo Chuang Hi-tech Material Co., Ltd., Wuhan, China). Table 1 shows the primary technical indices of all the original asphalts, residue after RTFO, and residue after PAV used in this study.

### 2.2. Test Method

#### 2.2.1. Aging Method

Five asphalts were aged using the RFTO (James Cox and Sons, Inc., Colfax, CA, USA) test by the Chinese specification JTGF40-2011 [25] to simulate short-term aging in mixing and placement. We used the PAV (Prentex, Dallas, TX, USA) test for in-service aging simulation following the procedure outlined in AASHTO M320 [26]. In PAV aging, the asphalts were first aged in the RTFO and then in the PAV at 300 psi for 20 h at 110 °C.

#### 2.2.2. Rheology Method

To analyze the effect of aging on the viscoelastic properties of HVA. DSR (Malvern Instruments Ltd., Malvern, UK) was used to test the high-temperature performance of the asphalts before and after aging; the temperature range was from 5 °C to 95 °C. Additionally, the low-temperature performance was conducted via BBR (Cannon Instrument Company, State College, PA, USA), and the temperature range was from ambient to −40 °C.

1. Temperature sweep test (TS)

TS of original, RTFO-, and PAV-aged asphalts was performed on a DSR with parallel-plate geometry (25 mm diameter, 1 mm gap) at a frequency of 10 rad/s and strain control mode. The tests were conducted from 40 °C to 80 °C.

According to the dynamic viscosity of the original, RTFO-, and PAV-aged asphalts at different temperatures obtained from the TS, the Arrhenius equation can be used to calculate the activation energy (*E_η_*) of each asphalt. The Arrhenius equation, shown in Equation (1), describes the mathematical relationship between the fluid’s dependence on temperature.

(1) $$\eta (T)=Ke^{\frac{{}_{E_{\eta }}}{RT}}$$

*T* is the temperature (Kelvin, K); η(*T*) is the viscosity at *T* (Pa·s); *K* is a material constant; *R* is the universal gas constant (*R* = 8.314 J·mol^−1^·K^−1^); and *E_η_* is the activation energy.

In studying the mechanical and engineering properties of asphalt materials, it is common practice to use appropriate coordinates to describe the temperature dependency of asphalt viscosity. This is achieved by fitting a linear equation and utilizing the slope of the line to characterize the temperature-sensitive properties of the asphalt material. To establish this linear relationship, we take the logarithm of both sides of Equation (1), resulting in Equation (2), which relates the logarithmic value of viscosity (*lg*(*η*)) to the reciprocal of temperature (1/*T*).

(2) $$\mathrm{lg}(\eta (T))=\mathrm{lg}(K)+\frac{E_{\eta }}{2.303RT}$$

To graphically represent the relationship between viscosity (*η*) and temperature (*T*), we can plot the values of 1/*T* on the horizontal axis and *η* on the vertical axis. By doing so, the slope of the resulting straight line will correspond to the coefficient value of 1/*T*, which is Eη2.303R. From this coefficient value, we can determine the *E_η_* of the asphalt material.

2. Repeated creep and recovery test (RCR)

In the study, RCR tests of original and RTFO residual asphalts were conducted on DSR. The specimens used were 1 mm thick and 25 mm in diameter. The RCRT consisted of 100 creep–recovery cycles, with each cycle having a creep time of 1 s followed by a recovery phase of 9 s. The entire test was conducted at a temperature of 60 °C, a stress level of 300 Pa, and a frequency of 1.59 Hz throughout the test.

3. Bending beam rheometer test (BBR)

BBR was conducted on PAV-aged asphalt samples to measure the asphalts’ low-temperature stiffness and relaxation properties via ASSHTO T313. The BBR test yielded two primary outcomes: the flexural creep stiffness, *S*(*t*); and the creep rate, m-value.

**Figure 1:**
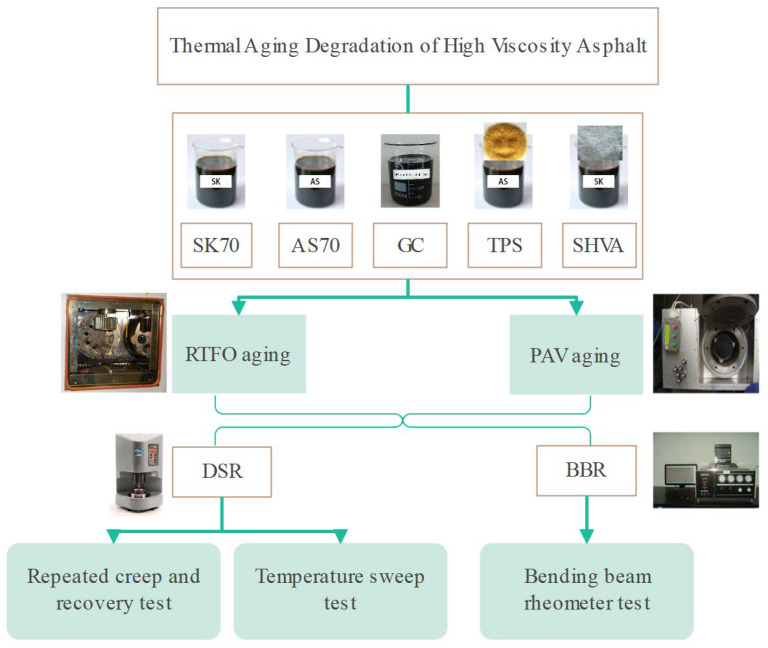
Flowchart of the experiments.

The experiments flowchart of this paper is summarized in Figure 1.

## 3. Results

### 3.1. Temperature Sweep Test (TS)

#### 3.1.1. Influence of Aging on Viscosity–Temperature Performance of HVA

**Figure 2:**
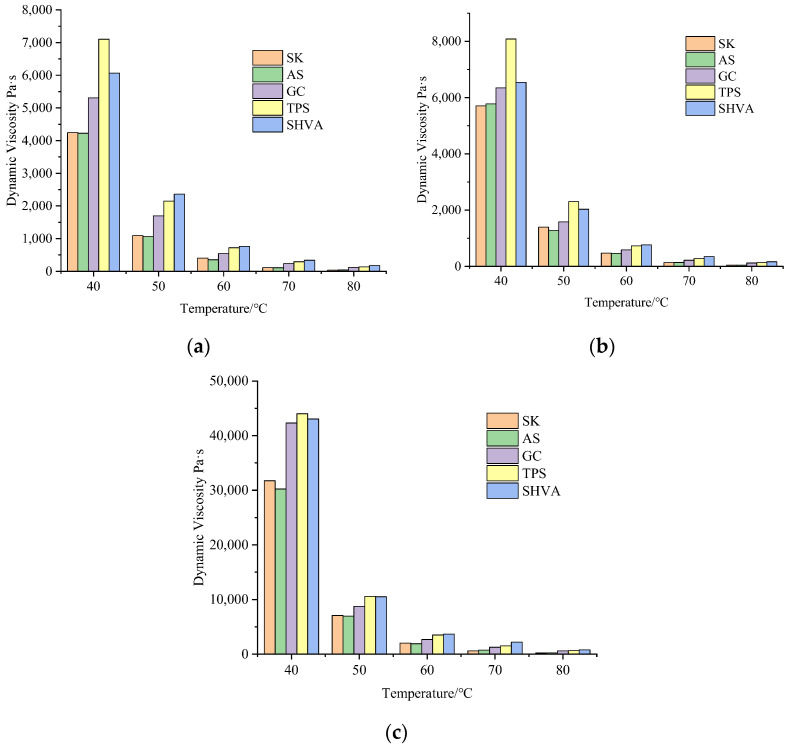
The change of dynamic viscosity with temperature under different aging states: (**a**) original; (**b**) RTFO; (**c**) PAV.

The results of the TS for both original and aged asphalts are presented in Figure 2. These tests were conducted to evaluate the impact of aging on the viscosity–temperature properties of HVA. As the temperature increased, the dynamic viscosity of both original and aged asphalts decreased sharply from 40 °C to 60 °C, followed by a slighter decrease. This behavior can be attributed to the increased thermal movement of asphalt molecules, which increases the distance between these molecules. Consequently, the intermolecular interactions are reduced, resulting in a decrease in viscosity [27]. Notably, the dynamic viscosity decrease rate differs between neat and modified asphalts for original and aged samples. For example, when the temperature increased from 40 °C to 50 °C, the dynamic viscosity decrease rates for original SK and AS were 74.3% and 74.9%, respectively; for RTFO-aged samples, they were 75.6% and 78%; and for PAV-aged samples, they were 77.7% and 77%. In comparison, the decrease rates for original GC, TPS, and SHVA were 68.0%, 69.8%, and 61.0%, respectively; for RTFO-aged samples, they were 75.1%, 71.5%, and 69.0%; and for PAV-aged samples, they were 79.3%, 76.0%, and 75.6%. Modified asphalts exhibited lower dynamic viscosity decrease rates compared to neat asphalts due to the presence of polymers within the asphalt. These polymers inhibit the movement of asphalt molecules, thereby increasing the resistance to deformation. Furthermore, it was observed that with increasing temperature, the viscosity decrease rate of all five asphalts gradually increased with aging, indicating that aging increases the temperature susceptibility of asphalts. Among the three modified asphalts, SHVA demonstrated the smallest decrease rate of viscosity, indicating its superior resistance to deformation under shear stress and lower temperature susceptibility.

During the RTFO aging process, chemical reactions occur in the asphalt, causing the breaking, reorganization, and polymerization of chemical bonds. These reactions inevitably lead to changes in viscosity, as they are closely related to the rheological properties of the asphalt. Compared to the original asphalts, the dynamic viscosity of the five asphalts increased after RTFO aging, as shown in Figure 2b. However, the rate of increase varied among the different asphalts: for SK and AS, the dynamic viscosity at 60 °C increased by 27.7% and 30.8%, respectively. The rise in asphaltene content is the main reason for the viscosity increase in neat asphalts [28]. On the other hand, for GC, TPS, and SHVA, the increase in dynamic viscosity after RTFO aging was only 8.0%, 0.9%, and 0.4%, respectively. This can be attributed to the modifiers’ degradation in the modified asphalts, which leads to a decrease in viscosity and an increase in fluidity. Furthermore, GC’s dynamic viscosity increase rate was 8.8 times and 20 times greater than that of TPS and SHVA, respectively. This indicates that the aging of modifiers has a much more significant effect on HVA’s viscosity than conventional SBS-modified asphalt. This difference may be attributed to the higher modifier contents present in HVA.

Figure 2c shows that after PAV aging, the dynamic viscosity of all five asphalts increases significantly. When compared to the original asphalts shown in Figure 2a, the dynamic viscosity of SK, AS, GC, TPS, and SHVA at 60 °C increased by 397.2%, 397.9%, 392.5%, 385.8%, and 382.1%, respectively. The increases in dynamic viscosity for the modified asphalts are slightly smaller than those of the neat asphalts. This indicates that with further degradation of the modifier, the aging inhibition effect of the modifier on the asphalts is substantially weakened. However, it is worth noting that the dynamic viscosity increase rate of the two HVA, TPS and SHVA, is 6.7% and 10.4% smaller than that of GC, respectively. This suggests that HVA still exhibits better anti-aging properties than ordinary SBS-modified asphalt even after long-term aging.

#### 3.1.2. Influence of Aging on *E_η_* of HVA

**Figure 3:**
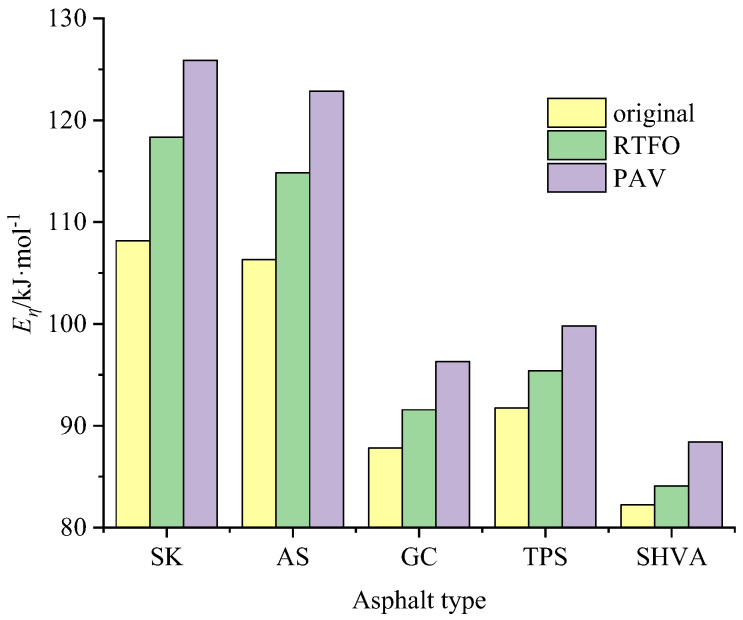
The *E*_η_ of asphalts under different aging states.

In Figure 2, the dynamic viscosity of each asphalt is measured at different temperatures under different aging methods. A regression analysis was conducted using the Arrhenius formula (Equation (2)) to analyze the data. The resulting regression equation and parameters are presented in Table 2. Based on these calculations, the *E_η_* can be determined and is illustrated in Figure 3.

Upon examining Figure 1 and Table 2, it can be observed that the viscosity of asphalt increases non-linearly as the aging process progresses from original to RTFO and PAV aging. However, when we plot the logarithmic value of viscosity (*lg*(*η*)) against the reciprocal of temperature (1/*T*), we observe a strong linear relationship. These linear relationships’ correlation coefficients (*R*^2^) are consistently greater than 0.98. The slope of the straight line represents the sensitivity of asphalt viscosity to temperature changes, while the magnitude of the slope reflects the difference in activation energy (*E_η_*). Comparing the same asphalt samples, we find that the slope of PAV-aged asphalts is much steeper than that of RTFO-aged asphalts. However, the slope magnitude difference between RTFO-aged and original asphalts is smaller. This suggests that, compared to PAV aging, the increase in *E_η_* due to RTFO aging is relatively small.

*E_η_* represents the minimum energy required by molecular chain segments to overcome the barrier and transition from their original position to nearby “holes” during the flow process of polymer materials. It serves as a parameter that describes the viscosity–temperature relationship of a material and reflects the difficulty of flow and the temperature sensitivity of asphalt. A larger *E_η_* indicates that the asphalt requires more minimum energy to initiate the transition, making it less sensitive to temperature changes [29].

Figure 3 shows that the *E_η_* values for SK and AS are significantly higher than those for GC, TPS, and SHVA. Specifically, the *E_η_* of SK or AS is approximately 1.23/1.21, 1.18/1.16, and 1.32/1.29 times greater than that of GC, TPS, and SHVA. This indicates that adding modifiers, such as polymers, to asphalts can significantly enhance their flexibility and reduce the energy required for flow transition. When comparing the *E_η_* values of the three modified asphalts in the same aging states, it can be observed that TPS has the highest *E_η_*, followed by GC, while SHVA has the lowest *E_η_*. However, the order of ZSV in Table 1 does not align with the *E_η_* values. SHVA has the highest ZSV, followed by TPS, and GC has the lowest ZSV. This discrepancy suggests that *E_η_* is not directly correlated with the viscosity of asphalt. Instead, *E_η_* is influenced by the molecular chain structure of the modifiers present in the modified asphalts rather than the total molecular weight. In the case of the two HVAs, TPS has a more rigid and polar molecular chain structure, resulting in a larger *E_η_*. On the other hand, SHVA has a more flexible linear molecular chain structure, leading to a smaller E*_η_*, indicating better temperature stability and workability.

It also can be seen in Figure 3 that the *E_η_* values of the five asphalts increase as the aging process deepens. This change in *E_η_* can directly reflect the alteration in the molecular structure of the asphalt. During RTFO, the neat asphalts primarily undergo thermal oxidation. This process targets the easily oxidizable functional groups in the branch chains of asphalt molecules, forming carbonyl groups. This oxidation reaction results in a significant increase in *E_η_*. For the modified asphalts, the RTFO aging mechanism in the asphalt phase is similar to that of the neat asphalt. However, the critical difference lies in the aging of the modifier. Generally, during the RTFO aging of modifiers, degradation and crosslinking reactions co-occur, but the degradation reaction is the dominant one [30]. This degradation reaction leads to the formation of medium-sized molecular structures within the modifier, which can reduce the *E_η_* of the asphalt. In long-term aging (PAV), as the oxidation reaction progresses, the main chains of asphalt molecules break, forming carbonyl groups and, in some cases, carboxyl groups with oxygen. This oxidation process weakens the temperature sensitivity of the asphalt and significantly increases the *E_η_* value.

#### 3.1.3. Influence of Aging on the Activation Energy Index (EAI) of HVA

The activation energy before and after aging is obtained to acquire the activation energy index, which can be used to react to the activation energy in the aging process, as seen in Equation (3):

(3) $$\mathrm{EAI}=\frac{E_{a,aged}-E_{a,unaged}}{E_{a,unaged}}\times 100\%,$$

where EAI is the activation energy index (%); *E_a_*_,*aged*_ is the activation energy after aging (kJ·mol^−1^); and *E_a_*_,*unaged*_ is the activation energy before aging (kJ·mol^−1^).

**Figure 4:**
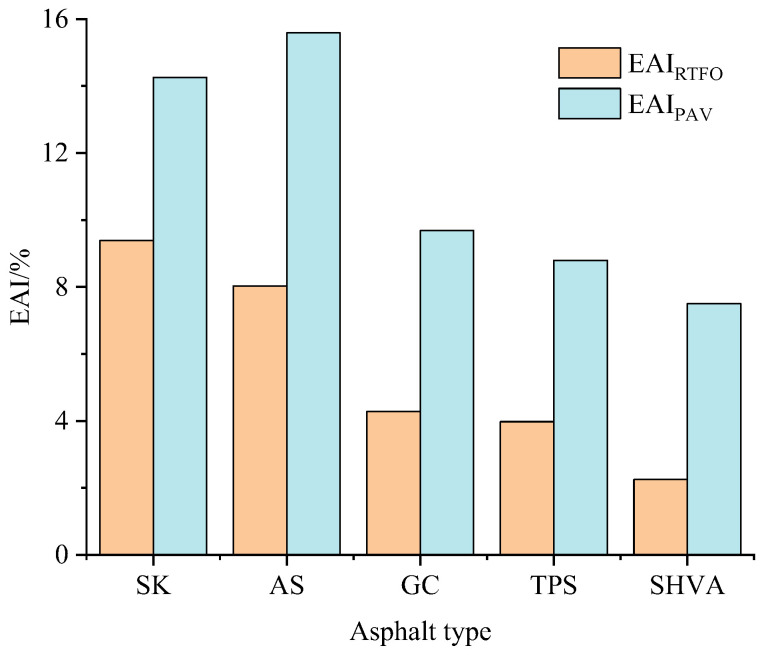
The EAI of five asphalts after different aging statements.

To further evaluate the anti-aging properties of the HVA, the EAI can be calculated using Equation (2). The EAI measures the difference in *E_η_* after aging, with a larger EAI indicating poorer anti-aging performance. The EAI values of the five asphalts after RTFO and PAV aging were calculated (EAI_RTFO_ and EAI_PAV_, respectively), as seen in Figure 4.

From Figure 4, it is evident that after aging, the EAI values of the two neat asphalts, SK and AS, are significantly higher than those of the three modified asphalts. For example, the EAI_RTFO_ of SK is 9.38%, which is 2.19, 2.36, and 4.17 times greater than GC, TPS, and SHVA, respectively. This indicates that adding modifiers in asphalts can greatly improve the short-term anti-aging performance of asphalt because the modifier in asphalts can inhibit the oxidation and polymerization reactions of asphalt. Comparing the two HVA, the EAI_RTFO_ values of TPS and SHVA are 3.98% and 2.25%, respectively. This suggests that SHVA exhibits better short-term anti-aging performance compared to TPS. Furthermore, considering the raw materials used, TPS is modified by AS, while SHVA is modified by SK. The contribution of the modifier in TPS to the reduction of EAI_RTFO_ is 4.05%, while for SHVA, it is 7.13%. This indicates that the contribution rate of the modifiers in SHVA against RTFO aging is higher than that of the modifiers in TPS; regarding EAI_PAV_, the values for SK, AS, GC, TPS, and SHVA increase to 16.37%, 15.59%, 9.68%, 8.80%, and 7.50%, respectively. This indicates that the long-term anti-aging performance, from best to worst, is SHVA, TPS, GC, AS, and SK. Additionally, for the two HVA, the contribution of the modifiers in TPS and SHVA to the reduction of EAI_PAV_ is 6.79% and 8.87%, respectively. The difference between EAI_PAV_ and EAI_RTFO_ for TPS and SHVA is 2.74% and 1.73%, respectively. This suggests that with aging depending, the contribution of the modifiers to anti-aging performance is significantly reduced, which leads to a deteriorated rheological property of HVA. Overall, the anti-aging performance of SHVA is better than that of the other asphalts, considering both short-term and long-term aging.

### 3.2. Repeated Creep and Recovery Test (RCR)

#### 3.2.1. Accumulated Stain (*r_acc_*)

**Figure 5:**
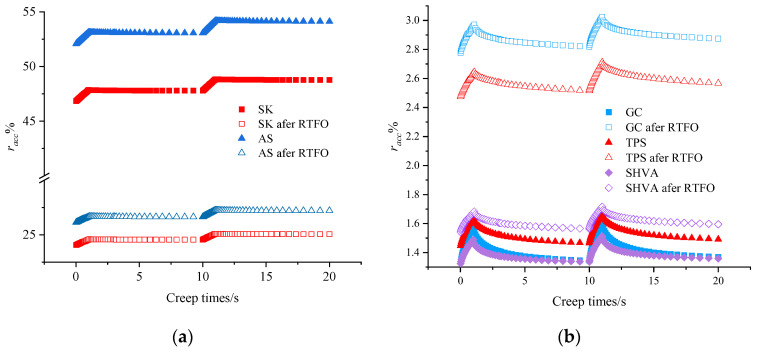
Evolution of *r_acc_* versus creep time before and after RTFO aging in the 50th and 51st cycles: (**a**) neat asphalts; (**b**) modified asphalts.

**Figure 6:**
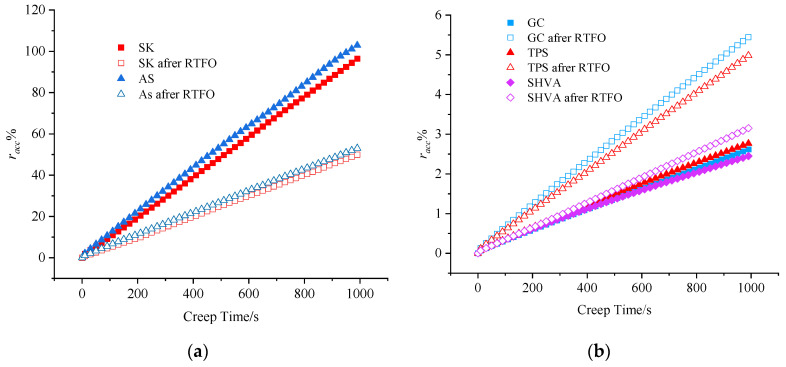
Evolution of *r_acc_* versus creep time before and after RTFO in the 100 cycles: (**a**) neat asphalts; (**b**) modified asphalts.

RCR is a method proposed to estimate the resistance to the accumulation of permanent strain in asphalt [31]. One of the critical indices from the RCR is the *r_acc_*, which is the sum of the residual deformation over multiple cycles. By comparing the *r_acc_* before and after aging, we can assess the aging effect on the deformation resistance and recovery ability of the asphalt. Figure 5 and Figure 6 illustrate the *r_acc_* of the asphalt before and after aging for the 50th~51st cycles and 100 cycles, respectively.

Based on Figure 5, it is evident that distinct differences exist in the deformation recovery behavior between neat asphalts and modified asphalt after RTFO aging. In the case of neat asphalt, the *r_acc_* curve shows a nearly straight line during the deformation recovery stage. This suggests that apart from a small amount of creep recovery and initial elastic recovery, the deformation recovery does not significantly change over time. It indicates that the deformation of neat asphalts during the creep stage is primarily due to flow deformation, and the permanent deformation generated after unloading is not recovered. On the other hand, for GC, TPS, and SHVA, the *r_acc_* initially decreases sharply and then gradually slows down during the deformation recovery stage. This indicates that the late-stage elasticity is continuously recovered. This can be attributed to the presence of elastomers in the polymer modifier molecules of the modified asphalt. These elastomers transfer their elastic properties to the asphalt, thereby enhancing the deformation recovery ability of the modified asphalt, especially at high temperatures.

It also can be seen in Figure 5 that the *r_acc_* of SK and AS after RTFO aging is significantly smaller than that before aging, while the *r_acc_* of GC, TPS, and SHVA is more significant than that before aging. In the case of neat asphalts, aging causes the volatilization of lightweight components, leading to the hardening of the asphalt. This hardening process enhances the elasticity of the asphalt, resulting in improved high-temperature performance. On the other hand, when modified asphalt undergoes RTFO aging, the degradation of the modifiers has a more significant impact than the aging effect on the neat asphalts. This degradation leads to an increase in the proportion of viscous components in the modified asphalts. As a result, the ratio of unrecoverable deformation in the asphalt increases, increasing *r_acc_*. After RTFO aging, the *r_acc_* of GC exhibited the highest growth of 109.85%, followed by TPS with a 72.05% increase, and SHVA with the smallest increase of 17.37%, compared to their values before aging. It can be inferred that the HVA exhibits better aging resistance than conventional SBS-modified asphalt (GC), and SHVA demonstrates the highest resistance to high-temperature deformation among the three types.

From Figure 6, it is evident that the *r_acc_* of each asphalt increases linearly with time, both before and after RTFO aging. The *r_acc_* of SK and AS is significantly larger than that of GC, TPS, and SHVA. This suggests that the deformation of neat asphalt is primarily due to viscous flow deformation, with minimal deformation recovery and a high accumulation deformation. This accumulated deformation upon unloading leads to permanent deformation.

After 100 cycles, the *r_acc_* of RTFO-aged SK and AS decreased by 49.9% and 50.0%, respectively, compared to the original asphalts. In contrast, the *r_acc_* of GC, TPS, and SHVA increased by 2.8%, 2.2%, and 0.7% after RTFO aging. This shows the existence of oxidation-induced hardening effect and degradation-induced softening effect. For neat asphalt, aging primarily involves the volatilization of light components and the hardening of the asphalt itself. However, in the case of modified asphalt, in addition to the aging of the asphalt, the aging degradation of polymer modifiers weakens the elasticity of the modified asphalt. This results in an increase in irrecoverable deformation under repetitive loading. Among the three modified asphalts, SHVA exhibits the smallest increase (0.7%) in *r_acc_* after RTFO aging, indicating that SHVA has excellent aging resistance.

From Figure 6b, the *r_acc_* curves of GC, TPS, and SHVA before RTFO aging are relatively close until 400 s. The slopes of these curves, in descending order, are GC, TPS, and SHVA, indicating that GC exhibits the fastest growth in residual deformation, followed by TPS, and SHVA shows the smallest increase. This suggests that the polymer modifier in SHVA has the highest elasticity and the strongest ability to resist repeated loading effects. After RTFO aging, the *r_acc_* curves of GC, TPS, and SHVA show significant differences compared to those before RTFO aging. The *r_acc_* curves of SHVA are located below those of GC and TPS, with the accumulated strains of GC and TPS (5.44% and 4.98%) after 100 cycles being 1.7 and 1.6 times greater than those of SHVA (3.15%), respectively. In summary, SHVA demonstrates better aging resistance compared to TPS and GC.

#### 3.2.2. Viscous Component of Binder Creep Stiffness (G_v_)

To minimize the impact of delayed elasticity on the test results, it is recommended to use the data from the 50th and 51st cycles of the RCR to fit the G_v_ index, which is suitable for characterizing the high-temperature performance of asphalt, especially modified asphalt. The G_v_ index is derived from the four-element Burgers model, a widely used model for describing the viscoelastic behavior of materials. The Burgers model combines a Kelvin model and a Maxwell model connected in series in normal and shear directions. This model is commonly employed to simulate creep and recovery measurements. The creep equation of the Burgers model can be seen in Equation (4):

(4) $$J(t)=\frac{\varepsilon (t)}{\sigma_{0}}=\frac{1}{G_{0}}+\frac{1}{G_{1}}[1-e^{-\frac{G_{1}}{\eta_{1}}t})]+\frac{t}{\eta_{0}}=J_{E}+J_{C}+J_{V},$$

where *ε*(*t*) is the total shear strain; σ_0_ is constant total stress (Pa); t is creep time (s); *G*_0_ is the spring constant of the Maxwell model (Pa); *G*_1_ is the spring constant of Kelvin model (Pa); *η*_0_ is viscosity coefficients of the Kelvin model (Pa·s); *η*_1_ dashpot constant of Kelvin model (Pa·s); *J_E_* is instantaneous elastic deformation compliance; *J_C_* is delayed elastic deformation compliance; and *J_V_* is viscous flow deformation compliance. G_v_ is 1/*J_V_* and is defined as the viscous component of the creep stiffness (MPa).

To capture the aging on the high-temperature performance characteristics of HVA, the data of the 50th and 51st cycles of RCR, seen in Figure 5, were used to fit the parameters of the four-element Burgers model, as described in Equation (4). The fitting parameters for each type of asphalt before and after RTFO aging are presented in Table 3. Additionally, Figure 6 displays the measured and simulated values of the creep compliance in the 50th cycle for the original and asphalts after the RTFO aging process.

**Figure 7:**
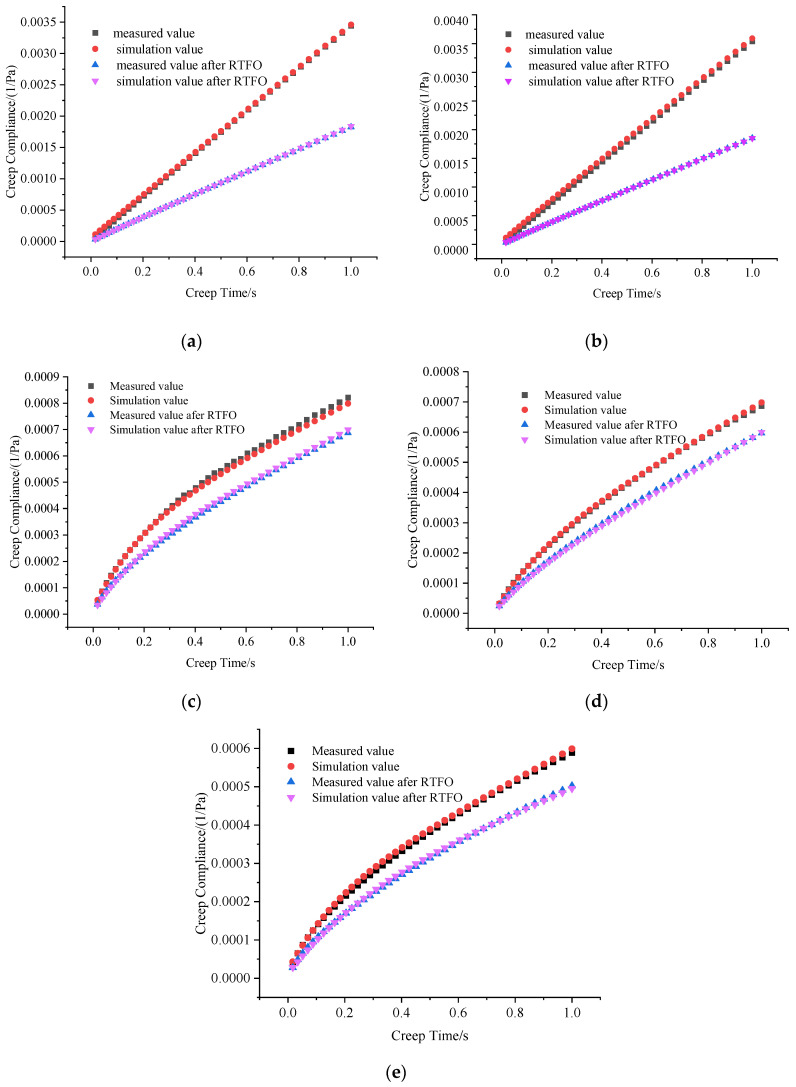
Measured and simulated value of original and RTFOT asphalts: (**a**) SK; (**b**) AS; (**c**) GC; (**d**) TPS; (**e**) SHVA.

The results depicted in Figure 7 demonstrate that the predicted creep compliance curves, both before and after RTFO aging, for the five types of asphalt closely match the measured curves. This indicates that the Burgers model can accurately simulate the creep behavior of asphalt.

After RTFO aging, the creep compliance of all five types of asphalt decreases compared to their respective values before aging. The creep compliance curves of SK and AS exhibit a linear increase with creep time, both before and after aging, indicating viscous flow deformation. On the other hand, the creep compliance curves of GC, TPS, and SHVA closely resemble logarithmic curves, indicating strong delayed elastic behavior. The creep compliance of GC, TPS, and SHVA, both before and after aging, is significantly lower than that of SK and AS. This is because the modifier used in GC, TPS, and SHVA imparts high elasticity to the asphalt, enhancing its deformation recovery at high temperatures. Furthermore, the creep compliance of the two HVA, TPS and SHVA, is notably lower than that of GC. This can be attributed to the higher crosslinking density of the modifier in HVA, resulting in minimum creep softness and stronger elasticity.

**Figure 8:**
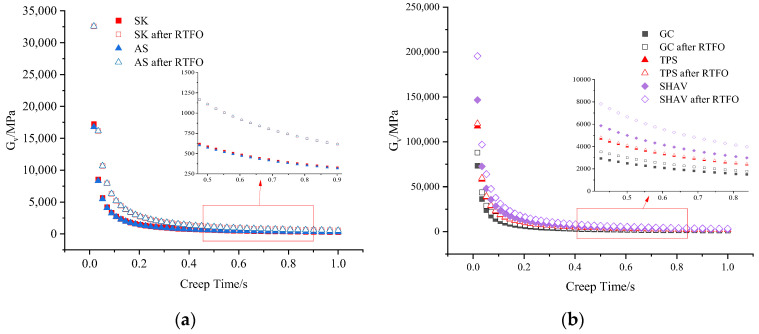
Evolution of G_v_ versus creep time for asphalts before and after RTFO aging: (**a**) neat asphalts; (**b**) modified asphalts.

Figure 8 shows that before aging, the G_v_ of each asphalt decreases rapidly with increasing creep time. The G_v_ values of SK and AS are nearly identical, indicating a minimal difference in their high-temperature performance. However, the G_v_ values of TPS and SHVA are notably higher than those of GC, suggesting that the two HVA exhibit superior high-temperature performance. SHVA has the highest G_v_ value among the three modified asphalts, indicating the best high-temperature performance. In Figure 8b, it can be observed that after aging, the trend of G_v_ with increasing creep time remains the same as before aging. Additionally, the G_v_ values after aging are slightly larger than those before aging. However, there is a noticeable difference in the G_v_ values of the three modified asphalts after aging. The G_v_ value of SHVA is significantly larger than that of TPS and GC, indicating that SHVA exhibits better aging resistance than GC and TPS. Furthermore, SHVA maintains high elasticity and deformation recovery ability even after aging, which aligns with the results obtained from the *r_acc_* analysis.

### 3.3. Bending Beam Rheometer Test (BBR)

**Figure 9:**
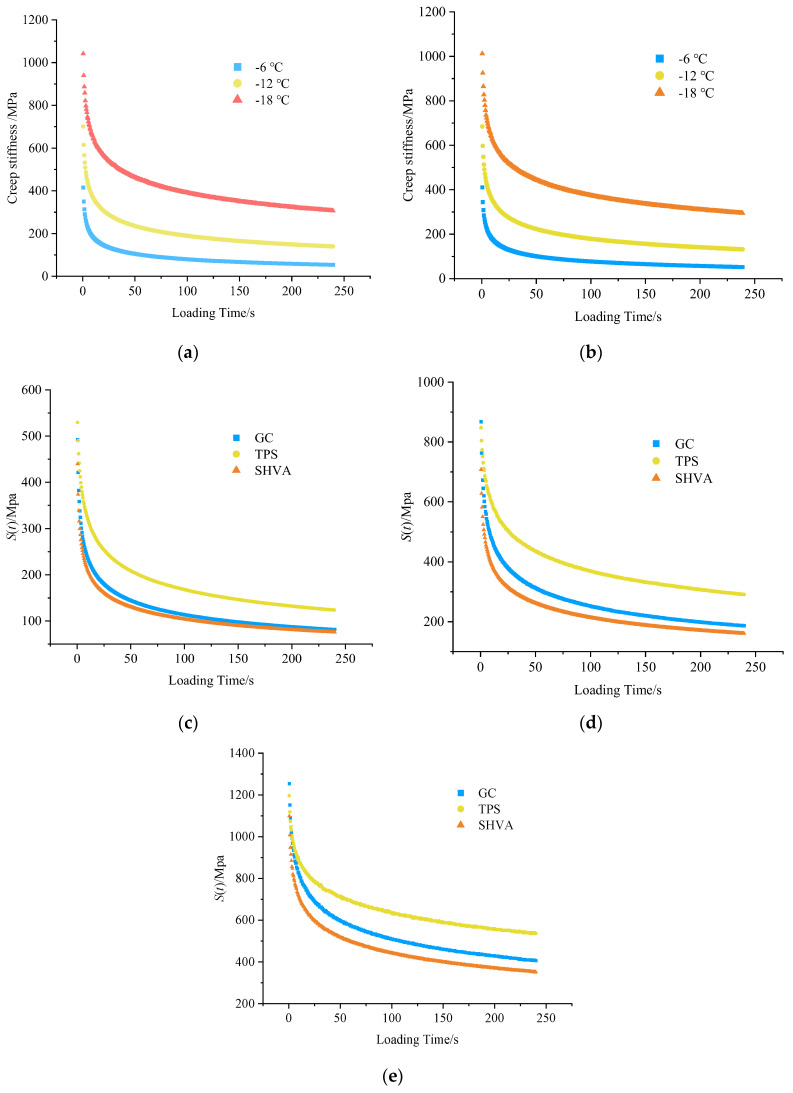
Variation of the creep stiffness (*S*(*t*)) with loading time for five asphalts at different temperatures: (**a**) SK; (**b**) AS; (**c**) −12 °C; (**d**) −18 °C; (**e**) −24 °C.

Figure 9 shows that the *S*(*t*) of the five asphalts initially decays rapidly during the loading period but gradually stabilizes as the loading time increases. Specifically, for the two neat asphalts, SK and AS, at experimental temperatures of −6 °C, −12 °C, and −18 °C, the loading times required for the *S*(*t*) to decay to 40% of its initial value were 13.5 s, 25.5 s, and 72.5 s for SK, and 13.5 s, 28 s, and 77.5 s for AS, respectively. Regarding the three modified asphalts, GC, TPS, and SHVA, at temperatures of −12 °C, −18 °C, and −24 °C, the loading times required for the *S*(*t*) of GC to decay to 40% of its initial value were 19 s, 35 s, and 107 s, respectively. Similarly, for TPS, the loading times were 19 s, 37.5 s, and 103 s, while for SHVA, the times were 19.0 s, 38.0 s, and 101.5 s, respectively. It is evident that as the test temperature increases, the *S*(*t*) decay occurs faster. This can be attributed to the higher temperature leading to increased creep compliance and more excellent resistance to deformation of the asphalt. However, it is essential to note that different asphalts exhibit varying *S*(*t*) decay rates at the same temperature. In particular, the three modified asphalts show a significantly faster decay rate than the two neat asphalts. For instance, at −18 °C, the loading time required for the *S*(*t*) of TPS to decay to 40% of its initial value is 51.5% that of SK and 48.4% that of AS, respectively. This indicates that the modified asphalt demonstrates better low-temperature performance than the neat asphalt after long-term aging. Furthermore, when comparing SHVA with GC and TPS at −24 °C, the loading time required for the *S*(*t*) of SHVA to decay to 40% of its initial value is 5.5 s and 1.5 s less than that of GC and TPS, respectively. This suggests that after PAV aging, the *S*(*t*) of SHVA experiences the fastest decrease, resulting in a higher rate of creep compliance and stronger resistance to low-temperature deformation.

**Figure 10:**
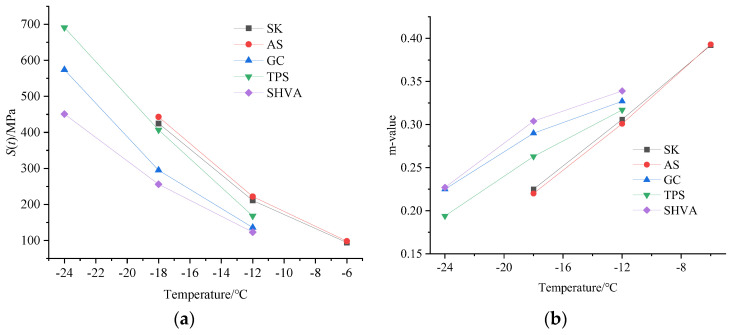
Test results of BBR: (**a**) *S*(*t*); (**b**) creep rate (m-value).

After long-term aging, the interaction force between asphalt particles increases, leading to increased stiffness and decreased relaxation ability under load. This results in severe degradation of the low-temperature performance of the asphalt. Figure 10 demonstrates that as the temperature drops, all five asphalts’ *S*(*t*) increases significantly while the m-value decreases noticeably. Generally, lower *S*(*t*) and higher m-values at low temperatures indicate better resistance to low-temperature cracking. Comparing the *S*(*t*) curves (Figure 10a), it is evident that AS and SK exhibit steeper slopes compared to the three modified asphalts. This suggests that AS and SK experience rapid *S*(*t*) growth as the temperature decreases, indicating insufficient resistance to low-temperature cracking. Among the three modified asphalts, the *S*(*t*) curve of SHVA is located at the bottom, meaning the slowest growth in stiffness as the temperature decreases. This implies that SHVA demonstrates the best resistance to low-temperature cracking. Analyzing the creep rate curves (Figure 10b), the order from bottom to top is AS, SK, TPS, GC, and SHVA, indicating that the low-temperature properties of the asphalts are ranked, in order of superiority, as SHVA, GC, TPS, SK, and AS. It is interesting to note that after long-term aging, the low-temperature performance of TPS is found to be worse than that of conventional SBS-modified asphalt GC. This suggests that the low-temperature performance of TPS deteriorates rapidly over time, which can be attributed to the aging of both the TPS high-viscosity modifier and the neat asphalt. Based on the material composition of the two HVA, it is observed that the *S*(*t*) of SK (base asphalt of SHVA) and AS (base asphalt of TPS) are 425 MPa and 423 MPa, and the m-values of SK and AS are 0.225 and 0.220 at −18 °C; the modifier in TPS and SHVA contributes to a reduction of creep stiffness at −18 °C by 36 MPa and 187 Mpa, respectively, and it contributes to an increase in the m-value of 0.043 and 0.084, respectively. These findings suggest that after long-term aging, the aging of base asphalt is the main reason for the changes in the low-temperature rheological properties of HVA. However, the modifier in SHVA exhibits better anti-long-term aging performance than the TPS modifier. For example, the contribution effect of the modifier in SHVA to the reduction of *S*(*t*) at −18 °C is 5.19 times greater than that of TPS. Similarly, the contribution effect of the modifier in SHVA to the increase in m-value at −18 °C is 1.95 times greater than that of TPS. This can be attributed to the modifier present in SHVA, which imparts high low-temperature flexibility.

## 4. Conclusions

In this study, five asphalts, including two neat asphalts (SK and AS), a conventional SBS-modified asphalt (GC), and two HVAs (TPS and SHVA), are chosen to comparatively investigate the effect of thermal aging on the rheology properties of HVA. RTFO and PAV aging were adopted to simulate the short-term and long-term aging of asphalts, respectively. The study specifically assessed the high-temperature rheological properties of SHVA through temperature sweep tests and RCR tests. Additionally, the low-temperature rheological properties were evaluated using the BBR test. The main conclusion can be drawn as follows:

(1) Aging increases the temperature sensitivity of asphalts significantly. According to the analysis of the temperature sweep test, compared with the SK, AS, and GC, both the original and aged HVAs (TPS and SHVA) showed lower temperature susceptibility and better resistance to deformation under shear stress at high temperatures (i.e., lower decreased rate of dynamic viscosity and more considerable dynamic viscosity at high temperatures).
(2) The *E_η_* of asphalts in different aging states can be calculated using the Arrhenius equation based on their viscosity at different temperatures. *E_η_* is a parameter that effectively characterizes asphalt’s rheological properties and temperature stability. The *E*_η_ of SHVA is smaller than that of TPS, indicating SHVA has better temperature stability and workability.
(3) EAI is a measure that directly reflects the aging resistance of asphalt. SHVA exhibits the best resistance to short-term and long-term aging, as indicated by the smallest values of EAI_RTFO_ (2.25%) and EAI_PAV_ (8.87%). The contribution of the modifiers in TPS/SHVA to the reduction of EAI_RTFO_ and EAI_PAV_ is 4.05%/7.13% and 6.79%/8.87%, respectively. This indicates that the contribution rate of the SHVA modifiers against RTFO and PAV aging is higher than that of the modifiers in TPS. The differences between EAI_PAV_ and EAI_RTFO_ for TPS and SHVA are 2.74% and 1.73%, respectively. This suggests that as the aging process progresses, the contribution of the modifiers to the anti-aging performance is significantly reduced, leading to a deterioration in the rheological properties of HVA.
(4) RTFO aging decreases the *r_acc_* of neat asphalts (SK and AS) by 49.9% and 50.0%, respectively, but increases the *r_acc_* of modified asphalts (GC, TPS, and SHVA) by 2.8%, 2.2%, and 0.7%, respectively, after 100 cycles. This shows the existence of the oxidation-induced hardening effect of neat asphalt and the degradation-induced softening effect of the modifier. SHVA possesses the smallest *r_acc_* (3.15%) and *r_acc_* increase rate (0.7%) after RTFO, demonstrating that SHVA had better aging resistance and high-temperature performance, which aligns with the results obtained from analyzing G_v_.
(5) After PAV aging, TPS demonstrates worse low-temperature performance compared to GC and SHVA. This is indicated by larger *S*(*t*) values and smaller m-values at the same temperature. The aging of the base asphalt is the primary factor contributing to the changes in the low-temperature rheological properties of HVA. Considering the material composition of SHVA, the modifier in SHVA exhibits a greater contribution to the low-temperature performance than TPS.

## Figures and Tables

**Table 1:** Basic technical indices of asphalts.

Asphalt Type	25 °C Penetration /0.1 mm	Softening Point/°C	5 °C or 10 °C Ductility/cm	60 °C ZSV/(Pa·s)	Toughness and Tenacity/(N·m)	Tenacity /(N·m)
SK	70.8	47.2	>100	-	-	-
AS	68.4	48.7	>100	-	-	-
GC	73.2	74.3	38.6	3246	15.1	6.8
TPS	40.1	90.8	35.6	20,436	30.6	20.2
SHVA	53.4	92.3	42.3	33,468	35.7	24.9
Residue after RTFO (163 °C, 5 h)						
SK	44.5	-	38.9	-	-	-
AS	39.7	-	39.2	-	-	-
GC	65.4	76.6	26.5	2098	18.3	7.4
TPS	36.5	94.5	20.5	11,256	32.4	20.8
SHVA	47.2	98.7	34.3	26,457	35.9	25.3
Residue after PAV (110 °C, 20 h)						
GC	39.4	-	14.7	1059	-	-
TPS	24.2	-	9.5	7659	-	-
SHVA	34.1	-	16.4	19,638	-	-

The ductilities of SK and AS were tested at 10 °C, the ductilities of the three modified asphalts and residues after RTFO were tested at 5 °C, and PAV was conducted at 10 °C.

**Table 2:** The Arrhenius regression equation and parameters of original, RTFO, and PAV-aged asphalts.

Asphalt Type		lg((*ηT*)) − 1/*T* Regression Equation	*K*	*R* ^2^
Original	SK	lg(*η*) = 5650.0/*T* − 14.413	3.86 × 10^−15^	0.9981
	AS	lg(*η*) = 5552.1/*T* − 14.119	7.60 × 10^−15^	0.9987
	GC	lg(*η*) = 4585.4/*T* − 10.959	1.10 × 10^−11^	0.9938
	TPS	lg(*η*) = 4791.2/*T* − 11.482	3.30 × 10^−12^	0.9975
	SHVA	lg(*η*) = 4357.8/*T* − 10.143	7.19 × 10^−11^	0.9958
RTFO	SK	lg(*η*) = 6184.2/*T* − 15.867	1.36 × 10^−16^	0.9991
	AS	lg(*η*) = 5998.4/*T* − 14.486	4.53 × 10^−16^	0.9979
	GC	lg(*η*) = 4781.7/*T* − 11.541	2.88 × 10^−12^	0.9894
	TPS	lg(*η*) = 4981.7/*T* − 12.041	9.10 × 10^−13^	0.9958
	SHVA	lg(*η*) = 4391.2/*T* − 10.252	5.60 × 10^−11^	0.9960
PAV	SK	lg(*η*) = 6574.7/*T* − 16.531	2.94 × 10^−17^	0.9987
	AS	lg(*η*) = 6417.5/*T* − 14.606	9.48 × 10^−17^	0.9985
	GC	lg(*η*) = 5029.3/*T* − 11.547	2.84 × 10^−12^	0.9804
	TPS	lg(*η*) = 5212.8/*T* − 11.995	1.01 × 10^−12^	0.9908
	SHVA	lg(*η*) = 4616.7/*T* − 10.192	6.43 × 10^−11^	0.9838

**Table 3:** The fitting parameters of the Burgers model for the original asphalts and asphalts after RTFO.

	Asphalt Type	SK	AS	GC	TPS	SHVA
Parameters						
G_0_/Pa	Original	100,000	100,000	50,000	111,111	50,000
	RTFO	50,000	50,000	100,000	142,857	100,000
η_0_/(Pa·s)	Original	294	286	1023	2000	2500
	RTFO	556	556	1500	2050	3333
G_1_/Pa	Original	20,000	25,000	3571	5235	5556
	RTFO	25,381	20,833	5263	10,753	5263
η_1_/(Pa·s)	Original	2500	3333	667	1111	1000
	RTFO	5000	5000	1000	2000	1250

## Data Availability

The raw and processed data required to reproduce these results are available by contacting the authors.

## Abbreviation

PA	porous asphalt pavement
HVA	high-viscosity asphalt
SBS	styrene-butadiene-styrene
SK	neat asphalt manufactured by SK Holdings, South Korea
AS	neat asphalt manufactured by ExxonMobil, Singapore
GC	styrene–butadiene–styrene modified asphalt manufactured by Hubei Guo Chuang Hi-tech Material Co., Ltd., China.
TPS	TAFPACK Super high-viscosity asphalt
SHVA	self-modified high-viscosity asphalt
RTFO	rolling thin-film oven
PAV	pressure aging vessel
TS	temperature sweep test
RCR	repeated creep and recovery test
MSCR	multiple-stress creep recovery
DSR	dynamic shear rheometer
BBR	bending beam rheometer
G*	complex shear modulus
δ	phase angle
*E_η_*	activation energy
ZSV	zero-shear viscosity
EAI	activation energy index
*r_acc_*	accumulated stain
G_v_	viscous component of binder creep stiffness
*S*(*t*)	flexural creep stiffness
m-value	creep rate
